# Resource Competition Triggers the Co-Evolution of Long Tongues and Deep Corolla Tubes

**DOI:** 10.1371/journal.pone.0002992

**Published:** 2008-08-20

**Authors:** Miguel A. Rodríguez-Gironés, Ana L. Llandres

**Affiliations:** Department of Functional and Evolutionary Ecology, Estación Experimental de Zonas Áridas (CSIC), Almería, Spain; University of Sydney, Australia

## Abstract

**Background:**

It is normally thought that deep corolla tubes evolve when a plant's successful reproduction is contingent on having a corolla tube longer than the tongue of the flower's pollinators, and that pollinators evolve ever-longer tongues because individuals with longer tongues can obtain more nectar from flowers. A recent model shows that, in the presence of pollinators with long and short tongues that experience resource competition, coexisting plant species can diverge in corolla-tube depth, because this increases the proportion of pollen grains that lands on co-specific flowers.

**Methodology/Principal Findings:**

We have extended the model to study whether resource competition can trigger the co-evolution of tongue length and corolla-tube depth. Starting with two plant and two pollinator species, all of them having the same distribution of tongue length or corolla-tube depth, we show that variability in corolla-tube depth leads to divergence in tongue length, provided that increasing tongue length is not equally costly for both species. Once the two pollinator species differ in tongue length, divergence in corolla-tube depth between the two plant species ensues.

**Conclusions/Significance:**

Co-evolution between tongue length and corolla-tube depth is a robust outcome of the model, obtained for a wide range of parameter values, but it requires that tongue elongation is substantially easier for one pollinator species than for the other, that pollinators follow a near-optimal foraging strategy, that pollinators experience competition for resources and that plants experience pollination limitation.

## Introduction

Deep corolla tubes and long tongues have evolved repeatedly and in different habitats. The Malagasy Star Orchid, *Angraecum sesquipedale*, and its pollinating moth, *Xanthopan morgani praedicta*, may be the most bizarre examples of this phenomenon, but they are by no means unique. Extremely long tongues have evolved repeatedly even within the Acherontiini hawkmoths [1]. Other examples of pollinators with disproportionally long tongues are some South African flies [2], nectar bats [3] and hummingbirds [4].

Darwin [5] postulated that long tongues select for deep flowers because (p. 202) plants that “compelled the moths to insert their probosces up to the very base, would be best fertilised.” He also suggested that corolla-tube elongation might itself select for pollinators with longer tongues, as there should be a positive correlation between tongue length and the amount of nectar that pollinators can extract from deep flowers. This arms-race interpretation was re-stated by Nilsson [6], whose experiments demonstrated that shortening of nectar spurs decreased reproductive success in *Platanthera bifolia* and *P. chlorantha*. These results have been replicated with *Disa draconis* [7] and *Gladiolus longicollis* [8].

The arms-race hypothesis is not the only mechanism that has been proposed to explain the evolution of deep corolla tubes. Wasserthal [9] suggests that corolla depth increases as plants adapt to a sequence of pollinators, each with a tongue longer than the previous one. According to the pollinator-shift model, the tongue length of pollinators does not increase in response to floral morphology. Tongue elongation takes place in a different ecological context, in response to unrelated factors (such as predation risk), and remains relatively constant while the flower deepens its corolla tube [9]. A recent phylogenetic analysis suggests that pollinator shifts are responsible for spur elongation in the columbine genus, *Aquilegia* [10].

Other authors have suggested that deep corolla tubes have evolved to exclude ineffective pollinators. As N. Muchhala points out (personal communication), this possibility was first suggested by Belt [11], who wrote: “but the structure of many [floral traits] cannot, I believe, be understood, unless we take into consideration not only the contrivances for securing the services of the proper insect or bird, but also the contrivances for preventing insects that would not be useful, from obtaining access to the nectar. Thus the immense length of the nectary of *Angraecum sesquipedale* of Madagascar might, perhaps, have been completely explained by Mr. Wallace, if this important purpose had been taken into account” (pg. 133). This idea was met with scepticism by Darwin who, in a note to the second edition of his book on orchid fertilisation [12], wrote: “I have no doubt of the truth of this principle, but it is hardly applicable here, as the moth has to be compelled to drive its proboscis as deeply down as possible into the flower” (pg. 165). Darwin's notwithstanding, the idea that long corolla tubes have evolved to prevent ineffective pollinators from reaching the nectar is still discussed [13]–[15]. Rodríguez-Gironés and Santamaría [16] used individual-based models to shows how this mechanism can operate. If there are short- and long-tongued pollinators competing for resources, and if there is variability in corolla-tube depth, short-tongued pollinators should concentrate their foraging effort on flowers with shallow corolla tubes, and long-tongued pollinators on flowers with deep corolla tubes [17]–[18]. Because optimal foraging leads to resource partitioning, there is selective fertilizing (assortative mating): pollen from flowers with shallow corolla tubes tends to end up in flowers with shallow corolla tubes, and pollen from flowers with deep corolla tubes tends to land on flowers with deep corolla tubes. Under these conditions, there is character displacement, and divergence in corolla-tube depth between the two plant species ensues [16].

In this paper, we extend the model of Rodríguez-Gironés and Santamaría [16] to study the conditions under which, if the two pollinator species have originally the same tongue length, natural selection can lead to the simultaneous divergence of corolla-tube depth and tongue length.

## Methods

We modelled the evolution of corolla depth in a community formed by two flower-visiting species (X and Y) and two plant species (A and B). For narrative simplicity, we refer to the flower-visitors as moths, but the model applies equally to any other taxa. (The two pollinating species need not be phylogenetically related. The model could be applied to a community with bird and bee pollinators.) Likewise, we refer to the nectar containers of flowers as corolla tubes, regardless of whether they are true corolla tubes or nectar spurs. This section starts with a qualitative description of the original model [16], and follows with detailed specification of the modifications made to study the co-evolutionary process. The reader is referred to the original paper for technical details that have not changed.

### Original model: non-evolving moths

We used an individual-based model (IBM) to simulate the evolution of corolla-tube depth in this community. The IBM approach allowed us to follow the fate of individual pollen grains and seeds, and to track the foraging success of each moth in each generation. Together with some assumptions concerning heritability and mutation rates, iterating the IBM allowed us to follow how corolla-tube depth changed through time. Moth tongue length was kept fixed in this model: half of the moths had short tongues and the other half had long tongues.

Flowering plants were located at the nodes of a 100×100 square grid. Because the optimal foraging strategy is quite complex [17]–[18], moths used a rule of thumb to implement a simplified version of this foraging strategy [16]. Moths moved at random in this grid. Upon encountering a plant, moths decided whether to land or keep on flying on the basis of the corolla-tube depth of its flowers. The moth strategy determined the probability of exploiting flowers of different corolla depth. Moths had a high probability of landing on plants with corolla-tube depth matching their proboscis, and the probability of landing decreased as the difference between corolla depth and proboscis length increased, although at the optimal foraging strategy long-tongued moths were less selective than short-tongued moths [16]–[18]. Upon landing on a plant, moths exploited flowers sequentially and left the plant when they encountered an unrewarding flower (see [16] for further details). The parameters of the rule of thumb could change in time (through random mutations and selection), allowing the population of pollinators to adjust their behaviour to changing conditions in the plant population.

When a moth was extracting nectar from a flower, there were certain probabilities that pollen was transferred from the flower's anthers to the body of the moth, and from the moth's body to the flower's stigmas. In most of our simulations, the probability that pollen was transferred from the moth to the flower depended on the plant and pollinator species involved, but it never depended on whether the pollinator's proboscis was longer or shorter than the flower's corolla tube [16]. The baseline model assumed that pollen was transferred from the moth to the plant with probability 0.3 for plant-moth species pairs (A, X) and (B, Y) and with probability 0.2 for species pairs (A, Y) and (B, X). The probability that pollen was transferred from the flower to the moth was assumed to be independent of the pollinator and flower involved in the interaction.

The pollen grains that arrived to a flower competed to fertilise its ovules and produce seeds. Mature seeds dispersed to neighbouring nodes and, from the seeds arriving to a node, one was selected to produce the plant that would grow the following generation. To penalise inbreeding, the genotype of a plant included a number of loci that could accumulate deleterious recessive mutations. The competitive ability of seeds decreased as the number of loci homozygote for the deleterious mutation increased.

The model assumed that coexistence of the two plant and pollinator species was assured by mechanisms having nothing to do with the pollination process: the number of moths of each species was fixed (population sizes might be limited by nesting sites) and the probability that a seed grew into a plant at a node depended on the proportion of plants of the same species during the last generation (seed predation might be frequency dependent).

### Co-evolutionary model: tongue length subject to selection

We now describe how the model was modified to study the possibility that deep corolla tubes and long tongues co-evolve. To allow for the evolution of proboscis length, the genome of moths must include a number of loci that determine proboscis length. There are two alleles for genes at these loci: the “zero” and the “one” alleles, and proboscis length (as corolla-tube depth for flowers) is determined by the number of “one” alleles. There are 15 such loci, so corolla-tube depth and proboscis length can take any integer value between 0 and 30. (Plants and moths are diploid.)

At the end of a foraging “season”, a payoff is assigned to each moth to determine its reproductive success. Payoffs are calculated as intake rate (amount of nectar collected divided by foraging time) minus the cost (per unit time) of maintaining the proboscis. There is no cost associated to proboscides shorter or equal than two units, and beyond this length the cost increases linearly with proboscis length. In the baseline model, the slope of the relationship between proboscis length and maintenance cost is 0.005 for pollinator species X and 0.100 for species Y.

While looking at the effect of nectar robbing on the evolution of deep corolla tubes, we noted that the deleterious mutations meant to penalise inbreeding played little role in the evolution of deep corolla tubes (unpublished results). To simplify the model, we therefore removed this part of the plants genome.

**Figure 1:**
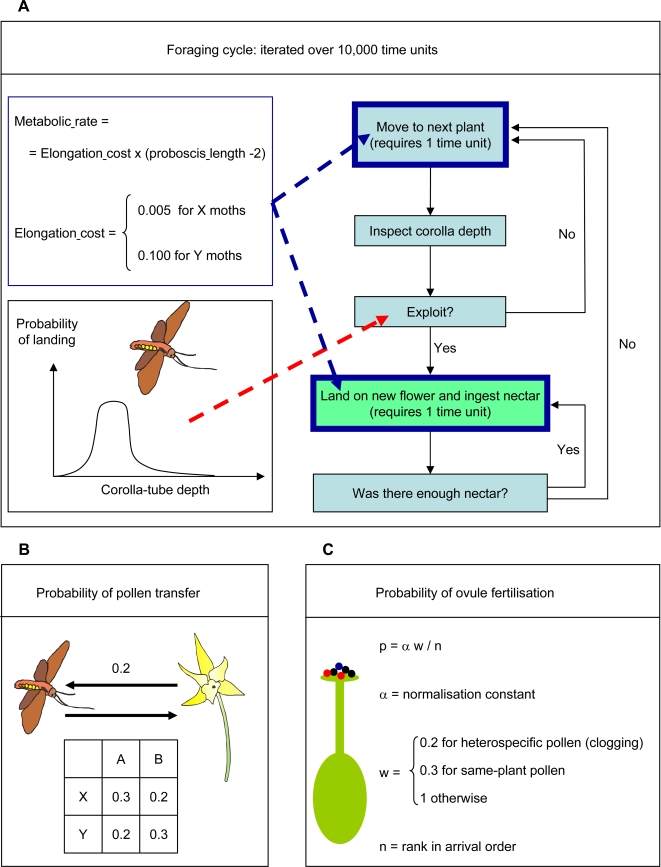
Schematic representation of the main model components. The foraging cycle (A) is iterated over 10,000 time units. Steps indicted in boxes with dark-blue outline require time, during which moths spend energy at a rate that increases with the length of their proboscis (as indicated in the box at the upper-left corner). The energy is recovered through nectar consumption (box with green background). The decision whether to exploit the flowers of a plant is probabilistic, and the probability of accepting a plant depends on the corolla depth of its flowers (box in the lower-left corner). When a moth exploits a flower, pollen can be transferred from the flower to the moth and from the moth to the flower, with different probabilities (B). At the end of the season, ovules are fertilised (C). The probability that a pollen grain fertilises an ovule depends on whether it arrived to the stigma early or late. Pollen grains from the same plant have a lower probability of fertilisation, and heterospecific pollen grains can prevent ovule fertilisation.

The implementation of the model requires assigning numeric values to a large number of parameters, all of which can potentially affect the evolutionary trajectories. To ensure that the results presented below are robust, we have assigned each parameter a “baseline value”, we have run the simulations with these values, and then we have selected small groups of parameters to change their values while the other parameters retained their baseline value. Fig. 1 provides a schematic representation of the main components of the model, together with the numerical values of the parameters in the baseline version of the model. For each set of parameter values, we have run ten simulations with different sequences of (pseudo) random numbers. Note that we study the effect of many parameters that were already explored in the original model: at the time, we were exploring the effect that these parameters had on the evolution of deep corolla tubes, while here we study the effect they have on the co-evolution between long tongues and deep corolla tubes.

## Results

### Co-evolution of Deep Corolla Tubes and Long Tongues

**Figure 2:**
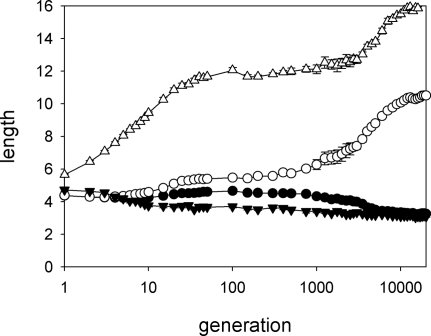
Evolutionary trajectories. Change in time of proboscis length (triangles) and corolla-tube depth (circles). The simulation was run ten times and, for each run, the mean values of proboscis length or corolla-tube depth were calculated for each species. In all figures, symbols represent the means (and bars the standard errors) of the ten species means.

There is an immediate divergence in the proboscis length of the two pollinator species (Fig. 2). Pollinators of species X (the species for which proboscis maintenance is less costly) rapidly increase the length of their proboscis, while there is a simultaneous, slight decrease in the proboscis length of species Y. These changes can be explained on purely economic terms: expected intake rate and metabolic costs are both increasing functions of proboscis length, so proboscis length stabilises at the point where the difference between intake rate and metabolic cost is maximised. Due to the differences between species in the cost of elongating the proboscis, X and Y moths differ in their optimal proboscis length. With the parameters of the model, the optimal proboscis length for X (Y) moths is greater (smaller) than the proboscis length at the beginning of the simulations.

**Figure 3:**
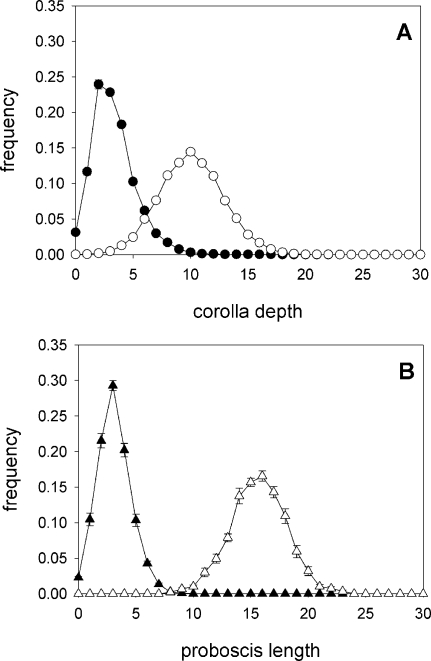
Equilibrium values. At evolutionary equilibrium (following 20,000 generations) (A) there is little overlap in the frequency distribution of corolla-tube depth of the two plant species and (B) virtually no overlap in the frequency distribution of proboscis length.

Plants take longer to respond. Flowers of species A, which are most effectively pollinated by moths of species X, have slightly deeper corolla tubes than flowers of species B after ten generations, but the difference is small and remains so for about 1000 generations, when the real divergence in corolla-tube depth takes place. The deepening of corolla tubes triggers a second phase of proboscis elongation in pollinators of species X. Following 20000 generations, evolution has essentially reached a plateau (Fig. 2–although the log scale of the figure is not the best to show this pattern), and there is virtually no overlap in the proboscis length of the two pollinator species and limited overlap in the depth of corolla tubes of flowers from A and B plant species (Fig. 3).

**Figure 4:**
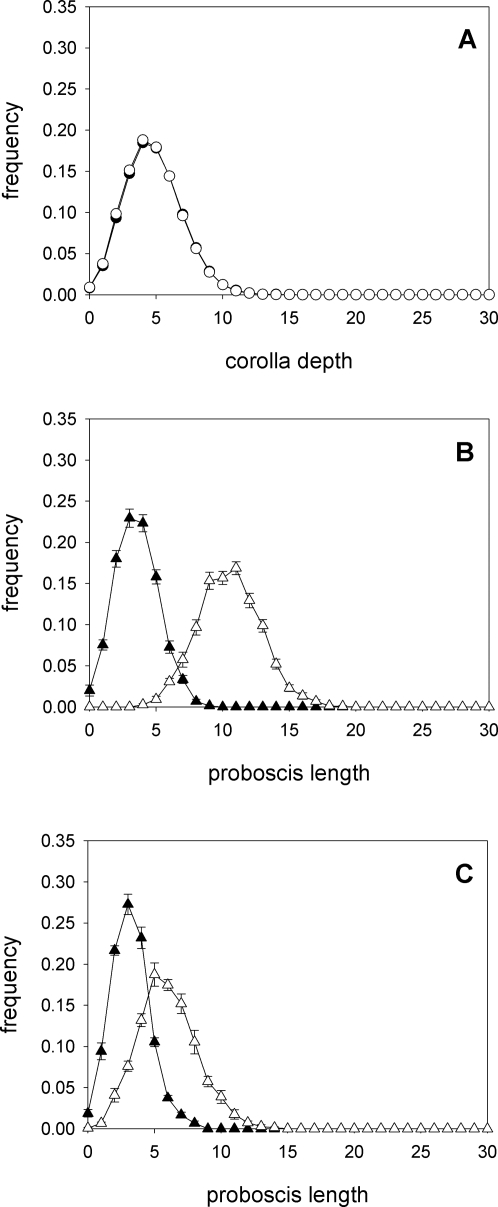
Evidence for co-evolution. (A) When the distribution of proboscis lengths is kept fixed, with no difference between the two moth species, corolla-tube depth does not evolve. (B) Proboscis length diverges when the distribution of corolla-tube depth is kept fixed, provided that there is variability in corolla-tube depth, but (C) there is hardly any divergence when all corolla tubes have the same depth.

We re-run the simulations preventing the evolution of flowers and moths to confirm that the evolutionary trajectories from Fig. 2 represent a co-evolutionary process. When we fix the proboscis length of pollinators (proboscis length is assigned to individuals at random, independently of the proboscis length of their parents, using the same probability distribution at each generation), there is absolutely no divergence in the depth of corolla tubes following 10000 generations (Fig. 4A). Preventing corolla-tube depth from evolving, however, does not halt the evolution of long proboscides (Fig. 4B). This may seem to imply that there is an intrinsic tendency among moths of species X to lengthen their proboscides, but this is not the case: moths are responding to the population variability in corolla-tube depth. When all flowers have the same corolla-tube depth (two units), there is basically no divergence among pollinator species in proboscis length (Fig. 4C). The observed difference in proboscis length when all flowers have the same corolla depth reflects a difference mutation-selection balance for the two pollinator species.

### Pollination Effectiveness

**Figure 5:**
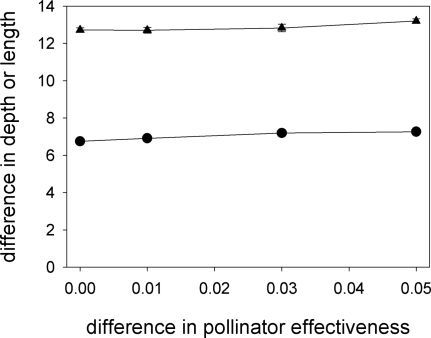
Pollination effectiveness. Asymmetries in pollination effectiveness (defined as per visit probability of pollen transfer) hardly affect the divergence of proboscis length (triangles) and corolla-tube depth (circles) after 20,000 generations.

**Figure 6:**
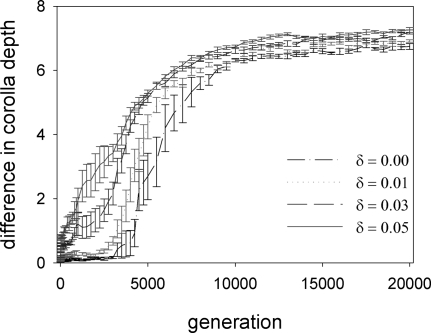
Pollination effectiveness and evolutionary rates. Asymmetries in pollination effectiveness (defined as per visit probability of pollen transfer) affect the speed of evolutionary change. When each moth species is a much better pollinator of one plant species than of the other (large *δ*), evolution proceeds much faster than when moths are equally good pollinators of the two plant species (*δ* = 0).

So far we have assumed that the probability of pollen transfer from the body of a moth of species J to a flower of species K, *p_JK_*, is *p_XA_* = 0.3, *p_XB_* = 0.2, *p_YA_* = 0.2 and *p_YB_* = 0.3. To study whether co-evolution requires the pairing, in terms of pollination effectiveness, of moth and plant species, we set *p_XA_* = *p_YB_* = 0.25+*δ* and *p_XB_* = *p_YA_* = 0.25–*δ* and run the simulations for different values of *δ*. Divergence in proboscis length and corolla-tube depth was observed for all values of *δ* (including *δ* = 0). The value of *δ* does not affect the differences in corolla-tube depth and proboscis length following 20000 generations (Fig. 5), but the rate of evolution does depend on *δ*: the lower the value of *δ*, the longer it takes for divergence in corolla-tube depth to get started (Fig. 6).

### Robustness of the Co-evolutionary Process

Co-evolution of corolla-tube depth and proboscis length is not an unlikely outcome that results from a careful choice of parameter values. Rather, co-evolution is observed with a wide range of parameter values.

**Figure 7:**
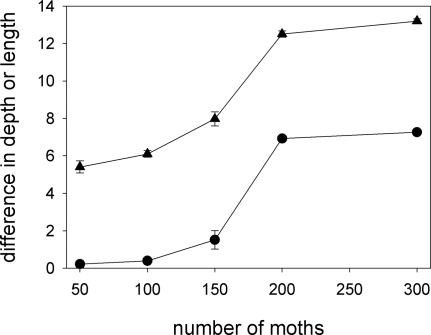
Moth population density. At low population densities of moths there is little divergence in proboscis length (triangles) and no divergence of corolla-tube depth (circles).

Decreasing the number of moths in the population from 300 to 200 has little effect on the co-evolutionary process, but a further reduction to 100 individuals or fewer leads to a marked reduction in the divergence between proboscis length of the two pollinator species, and no differentiation whatsoever in the depth of corolla tubes of the two plant species (Fig. 7). In the simulations with 50 or 100 moths, there is no hint that proboscis length or corolla-tube depth is evolving in any species after 20,000 generations. The lack of divergence in corolla-tube depth with these parameter values cannot be attributed to a delayed response.

**Figure 8:**
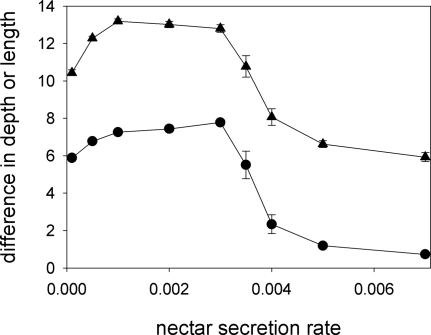
Nectar secretion rate. While proboscis length diverges for all values of nectar secretion rate (triangles), divergence of corolla- -tube depth is only observed for low and intermediate values of nectar secretion rate (circles).

Co-evolution between proboscis length and corolla-tube depth takes place for low and intermediate values of nectar secretion rate (Fig. 8). When nectar production is very high, moth species differ in their proboscis length, but plant species do not differ in their distribution of corolla-tube depth. This is because, at very high nectar secretion rates, long-tongued pollinators visit all flowers they encounter [18] and “short tongued” pollinators can track the increases in corolla-tube depth. Short-tongued pollinators can exploit all available flower types, and there is no resource partitioning.

**Figure 9:**
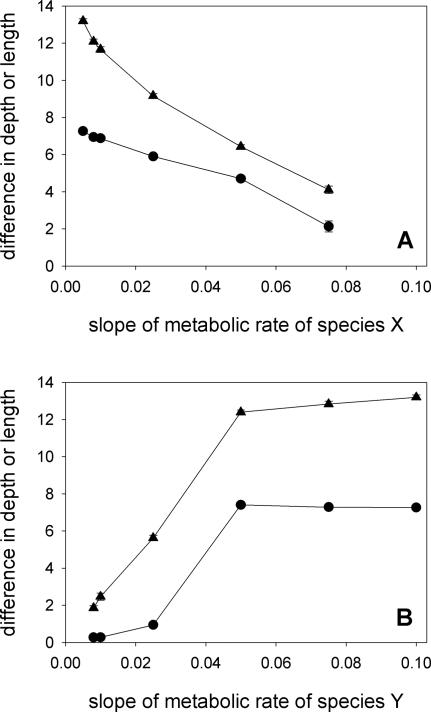
Cost of increasing proboscis length. The model assumes a linear relationship between the cost of producing a proboscis and its length. For the baseline model, the slope of this relationship is 0.05 for X moths and 0.1 for Y moths. Divergence of proboscis length (triangles) and corolla-tube depth (circles) disappears when the cost of producing a proboscis of a given length is equal for the two moth species, whether (A) we increase the cost for species X letting the cost for species Y fixed, or (B) we decrease the cost for species Y letting the cost for species X fixed.

**Figure 10:**
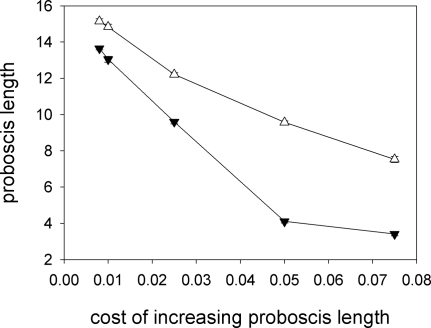
The relationship between maintenance cost and equilibrium proboscis length depends on whether the competitor moth species has higher (empty triangles) or lower (black triangles) maintenance cost.

In the baseline model, maintenance of long proboscides is less costly for moths of species X than for moths of species Y. As a result, moths of species X quickly develop very long proboscides (Fig. 2). When the cost of maintaining long proboscides is similar for the two species (either because we increase the cost to species X, Fig. 9 A, or because we decrease the cost to species Y, Fig. 9B), the difference between proboscis length at evolutionary equilibrium for the two moth species decreases. This, in turn, has an effect on the evolution of corolla-tube depth: when the two moth species have similar proboscis lengths, there is little divergence in corolla-tube depth (Fig. 9). It is interesting to note that there is not a one-to-one relationship between maintenance cost and equilibrium proboscis length. Consider an intermediate value of maintenance cost, say 0.04. We have run a set of simulations where the maintenance cost for species X was 0.04 and for species Y 0.1, and another set where maintenance costs for X and Y where 0.005 and 0.04. If maintenance cost fully determined the equilibrium value of the proboscis length, X moths in the first set of simulations would have the same proboscis length as Y moths in the second set. This, however, is not the case. The equilibrium proboscis length is shorter when the other species has a lower maintenance cost (and hence a longer proboscis) than when the other species has a greater maintenance cost (and hence a shorter proboscis, Fig. 10).

We obtained the same results when we changed the values of the following parameters: the probability that pollen was transferred from the flower to the pollinator, the probability of stigma clogging by pollen from flowers of different species and the relative competitive strength, during fertilisation, of pollen from the same or different (co-specific) plants (data not shown).

### Perceptual Errors

The baseline model assumes that pollinators can assess without errors the depth of the corolla tubes and that they use this information to decide whether to land on the plants they encounter. It seems safe to assume that pollinators are unable to assess accurately the depth of the corolla tubes. This, however, does not necessarily preclude the evolution of deep corolla tubes: even moths visiting plants irrespectively of their floral traits may sample more flowers with their preferred corolla-tube depth than with less preferred corolla-tube depths, provided that they use an appropriate giving-up rule. The bias introduced by such rules, however, will depend on the number of flowers per plant: if there is a single flower per plant, there will be no difference between sampling plants and flowers; if there are 1,000 flowers per plant and moths leave the plants after probing a single flower when they encounter a less preferred corolla-tube depth, essentially all the flowers they visit will have their preferred corolla-tube depth. Because perceptual errors affect the probability of landing on a plant, but not the decision whether to leave it or not (which is contingent on the amount of nectar encountered), when studying the effect of perceptual errors on the co-evolution of long proboscides and deep corolla tubes we must consider the interaction of two factors: the number of flowers per plant and the accuracy with which moths can assess corolla depth.

To introduce perceptual inaccuracies, we assume that a corolla depth *d* is perceived by moths as *δ* = *d*+*ε*, where *ε* is a random deviate, normally distributed, with mean 0 and standard deviation *γ* *d*. The magnitude of the perceptual error is therefore assumed proportional to the size of the stimulus, in agreement with psychophysical findings [19]. For vertebrates, the coefficient of variation of the error term, *γ*, is typically of the order of 0.2 [19]. Because moths don't have access to the real depth of the corolla tubes, they must decide whether to land on a plant or not using the perceived depth, *δ* [16].

**Figure 11:**
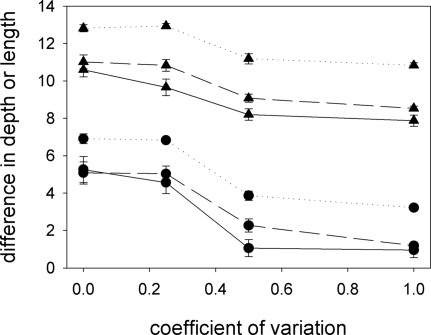
Perceptual errors. As the magnitude of perceptual errors (indicated by the coefficient of variation of the noise term) increases, equilibrium differences in proboscis length (triangles) and corolla-tube depth (circles) decrease. The effect is more pronounced when there are few flowers per plant (solid line = one flower per plant; dashed line = two flowers per plant) than when each plant has several flowers (dotted line = five flowers per plant).

Both the magnitude of the perceptual errors and the number of flowers per plant affect proboscis length and corolla-tube depth after 20,000 generations. Divergence in these traits is greater when there are several flowers per plant and when assessment of corolla-tube depth is accurate (Fig. 11). When there are five flowers per plant, corolla-tube depth diverges even in the presence of large levels of perceptual errors (*γ* = 1.0), but with one or two flowers per plant, divergence of corolla-tube depth disappears with large perceptual errors (*γ* = 0.5, Fig. 11).

### Pollination Limitation

We have so far assumed that each flower has two pollinaria. Increasing the number of “pollen parcels” from two to ten, while keeping all other parameter values fixed, interferes with the divergence of corolla depths between the two plant species. In the baseline model, the mean corolla-tube depth following 20.000 generations was 7.26±0.11 (mean±standard error of ten runs) units longer in one species than in the other. The difference is reduced to 1.95±0.64 units when the two pollinaria are transformed in ten pollen parcels. This, however, does not mean that long corolla tubes can only evolve in systems where plants have two pollinaria. Reducing the lifespan of flowers to one tenth of the baseline value, so that the average number of visits per flower is reduced from 20 to 2, leads to a further decrease in the divergence of corolla-tube depth (0.24±0.08 units). With the short life span of flowers, however, there is little time for nectar to accumulate in deep corolla tubes. In this scenario moths tend to find either empty flowers or flowers that have the amount of nectar they had at the time of opening, assumed to be constant in the baseline model. As a result, long-tongued pollinators gain little from specialising on flowers with deep corolla tubes. Rather, moths tend to visit every flower they encounter and deep corolla tubes do not evolve. It is often the case, however, that flowers with deep corolla tubes secrete more nectar than flowers with short corolla tubes. We can introduce this correlation in the model assuming that, at the time of opening, the nectar column reaches one fourth of the corolla tube depth. With this assumption, long-tongued moths gain from specialising on deep flowers and long corolla tubes readily evolve (9.12±0.13 units). These results suggest that pollination limitation is a pre-requisite for the evolution of long corolla tubes.

## Discussion

In order to explain the co-evolution of long proboscides and deep corolla tubes, we must understand the evolutionary forces behind two processes: proboscis elongation and deepening of corolla tubes. There are at least four putative mechanisms for the evolution of flowers with deep corolla tubes: increased pollination effectiveness through improved contact between flower and pollinator [5]–[6], promotion of flower constancy if specialising on a single flower type increases the foraging efficiency of pollinators [20], exclusion of ineffective pollinators [11], [13]–[14] and character displacement if pollinators are optimal foragers [16]. Proboscis elongation has been explained in two ways: it will result if it increases the foraging efficiency of pollinators [6] and if it decreases their predation risk [9].

The idea that character displacement, due to the foraging strategies of pollinators, promotes divergence in corolla-tube depth is related to two of the previously proposed hypotheses: that deep corollas evolve to promote flower constancy [20] and that they contribute to exclude unwanted visitors [13]–[14]. Laverty [20] suggested that pollinators might need different skills to exploit the nectar from flowers with different structures, and that learning to exploit one type of flowers might interfere with the possibility of becoming proficient at other flower types. If pollinators specialised on flowers with deep corolla tubes became inefficient at exploiting flowers with shallow corolla tubes, divergence of corolla-tube depth would indeed promote flower constancy. Laverty [20], however, found little empirical support for this idea: learning to extract nectar from a tubular corolla does not seem to constraint the ability of pollinators to exploit flowers with shallow corolla tubes. Rodríguez-Gironés and Santamaría [16] also argue that divergence in corolla-tube depth will promote flower constancy, but for a different reason: when there is variability in the corolla-tube depth of flowers and in the proboscis length of pollinators in a community, and assuming that there is resource competition, optimal-foraging pollinators will specialise in flowers with matching corolla-tube depths [17]–[18].

The mechanism proposed by Rodríguez-Gironés and Santamaría [16] is also related to the idea of excluding ineffective pollinators [13]–[14]. Excluding ineffective pollinators seems to imply that some pollinators are intrinsically better than others at transferring pollen from the flower to the pollinator and back to the next flower. It suggests that pollinators differ in their degree of “mechanical fit” with the flower. As shown by Rodriguez-Gironés and Santamaría [16] and corroborated by the present model, this need not be the case. Co-evolution between corolla tube depth and proboscis length persists when the per-visit probability of pollen transfer is the same for all plant-pollinator combinations. In this scenario, what makes some pollinators more effective than others at transporting pollen from one flower to a co-specific flower is their foraging strategy: nectar feeders are effective pollinators of their preferred flowers. But the foraging strategy of an optimally-foraging pollinator is context dependent: whether a given moth is a poor or an effective pollinator of a flower type will depend on the distribution of corolla-tube depths and proboscis lengths in the community.

We see no reason to assume that a single combination of hypotheses should explain all known examples of co-evolution between long proboscides and deep corolla tubes nor, for that matter, that all systems where a long-tongued visitor pollinates flowers with deep corolla tubes are the result of a co-evolutionary process. Each species has been subject to its particular evolutionary history and different mechanisms may lie behind the evolution of similar structures in different species. For example, Wasserthal's [9] suggestion that deep corolla tubes evolve after pollinator shifts is not quite a co-evolutionary explanation and requires as starting point a complex community, with short- and long-tongued pollinator species. It is therefore important to understand which ecological scenarios will allow the different mechanisms to evolve.

In the present paper, we have examined the conditions under which long proboscides and deep corolla tubes can co-evolve assuming that the driving force behind the deepening of corolla tubes is character displacement [16] and that pollinators with longer proboscides can obtain more nectar in flowers with deep corolla tubes [5]–[6]. Although this pair of hypotheses does lead to the co-evolution of deep corolla tubes and long proboscides under a wide range of ecological scenarios, such co-evolution is not a universal outcome. In particular, we can identify certain requirements for co-evolution to take place. (1) The cost of maintaining a proboscis of a given length must be different for the two moth species (Fig. 9). If the cost is similar for the two species, proboscis length does not diverge, individual moths do not specialise on flowers with one corolla-tube depth, there is no selective fertilizing within plants and there is no evolutionary pressure for divergence in corolla-tube depth. (2) Nectar must be a limiting resource (Figs. 7 and 8). In the absence of resource competition there is little divergence in proboscis length (Figs. 7 and 8) and, even if proboscis length did diverge, pollinators would tend to forage at random, because there would be no benefit to being selective [18]. (3) Pollinators must be able to forage selectively on flowers with certain corolla-tube depths. This will be the case if they can accurately assess corolla-tube depth before landing or, if they have limited perceptual capabilities, if there are enough flowers per plant that the giving-up rule ensures selectivity at the flower level even if there is no selectivity at the plant level (Fig. 11). (4) Pollination must be a limiting factor for plant reproductive success. Flowers with deep corolla tubes only evolved if each flower could export a maximum of two sets of pollen grains or if flowers received few pollinator visits.

The results of the simulations in the presence of perceptual errors could be used to answer Darwin's claim that parasite avoidance could not explain the evolution of long corolla tubes [12]. If our understanding of his note is correct, Darwin was claiming that once the moth has attempted to drink the nectar, it is irrelevant for the plant whether it succeeded or not, because pollen will have already been removed or deposited. Even if we ignore the fact that visit duration increases with the amount of nectar available to the moth, and that more pollen can be transferred in longer visits, the fact is that short-longed pollinators will visit fewer flowers with deep corolla tubes. Once again, it is the foraging strategy of pollinators which ultimately drives the evolution of long corolla tubes.

We have so far used IBMs to study the evolution of deep corolla tubes [16] and the coevolution between deep corolla tubes and long proboscides. The next step will be to investigate whether pollinators can promote sympatric speciation.
